# West Nile Virus and Other Domestic Nationally Notifiable Arboviral Diseases — United States, 2019

**DOI:** 10.15585/mmwr.mm7032a1

**Published:** 2021-08-13

**Authors:** Grace M. Vahey, Sarabeth Mathis, Stacey W. Martin, Carolyn V. Gould, J. Erin Staples, Nicole P. Lindsey

**Affiliations:** ^1^Arboviral Diseases Branch, Division of Vector-Borne Diseases, National Center for Emerging and Zoonotic Infectious Diseases, CDC; ^2^Epidemic Intelligence Service, CDC.

Arthropod-borne viruses (arboviruses) are transmitted to humans primarily through the bites of infected mosquitoes and ticks. West Nile virus (WNV) is the leading cause of domestically acquired arboviral disease in the United States (*1*). Other arboviruses, including La Crosse, Jamestown Canyon, Powassan, eastern equine encephalitis, and St. Louis encephalitis viruses, cause sporadic disease and occasional outbreaks. This report summarizes surveillance data for nationally notifiable domestic arboviruses reported to CDC for 2019. For 2019, 47 states and the District of Columbia (DC) reported 1,173 cases of domestic arboviral disease, including 971 (83%) WNV disease cases. Among the WNV disease cases, 633 (65%) were classified as neuroinvasive disease, for a national incidence of 0.19 cases per 100,000 population, 53% lower than the median annual incidence during 2009–2018. More Powassan and eastern equine encephalitis virus disease cases were reported in 2019 than in any previous year. Health care providers should consider arboviral infections in patients with aseptic meningitis or encephalitis, perform recommended diagnostic testing, and promptly report cases to public health authorities. Because arboviral diseases continue to cause serious illness, and annual incidence of individual viruses continues to vary with sporadic outbreaks, maintaining surveillance is important in directing prevention activities. Prevention depends on community and household efforts to reduce vector populations and personal protective measures to prevent mosquito and tick bites such as use of Environmental Protection Agency–registered insect repellent and wearing protective clothing.*^,^^†^

Arboviruses are maintained in transmission cycles between arthropods and vertebrate hosts, including humans and other animals (*2*). Humans primarily become infected when bitten by an infected mosquito or tick. Most human arboviral infections are asymptomatic; symptomatic infections commonly manifest as systemic febrile illness, similar to bacterial or parasitic diseases transmitted by ticks, and less commonly as neuroinvasive disease.

Most endemic arboviral diseases are nationally notifiable and reported by state health departments to CDC through ArboNET, the national arboviral surveillance system managed by CDC and state health departments, using standard surveillance case definitions that include clinical and laboratory criteria (*3*). Cases are reported by patient’s state and county of residence. Confirmed and probable cases were included for 2019. Cases reported as meningitis, encephalitis, acute flaccid paralysis (AFP), or unspecified neurologic presentation were classified as neuroinvasive disease; the remainder were considered nonneuroinvasive disease. Incidence was calculated using U.S. Census 2019 midyear population estimates and reported neuroinvasive disease cases, which are more reliably diagnosed and reported than nonneuroinvasive disease cases because of the associated morbidity.

A total of 1,173 cases of domestic arboviral disease were reported for 2019; cases were caused by the following viruses: West Nile (971 cases; 83% of all cases), La Crosse (55; 5%), Jamestown Canyon (45; 4%), Powassan (43; 4%), eastern equine encephalitis (38; 3%), St. Louis encephalitis (17; 1%), and unspecified California serogroup (four; <1%). Cases were reported from all states except Delaware, Hawaii, and Vermont, and from 380 (12%) of the 3,142 U.S. counties. Overall, 802 (68%) domestic arboviral disease cases were classified as neuroinvasive.

The 971 WNV disease cases were reported from 285 counties in 43 states and DC; 633 (65%) cases were neuroinvasive, and 794 (82%) patients had illness onset during July–September (Table 1). The median patient age was 60 years (interquartile range [IQR] = 46–70 years); 572 (59%) were male. A total of 662 (68%) patients were hospitalized, and 60 (6%) died.

**TABLE 1:** Number and percentage of reported cases of West Nile virus and other arboviral diseases (N = 1,173), by virus type and selected patient characteristics — United States, 2019*

Characteristic	Virus type, no. (%)^†^					
	West Nile (n = 971)	La Crosse (n = 55)	Jamestown Canyon (n = 45)	Powassan (n = 43)	Eastern equine encephalitis (n = 38)	St. Louis encephalitis (n = 17)
**Age group, yrs**						
<18	27 (3)	51 (93)	2 (4)	5 (12)	4 (11)	0 (—)
18–59	445 (46)	1 (2)	21 (47)	9 (21)	10 (26)	5 (29)
≥60	499 (51)	3 (5)	22 (49)	29 (67)	24 (63)	12 (71)
**Sex**						
Male	572 (59)	33 (60)	30 (67)	31 (72)	27 (71)	12 (71)
Female	399 (41)	22 (40)	15 (33)	12 (28)	11 (29)	5 (29)
**Period of illness onset**						
Jan–Mar	7 (1)	0 (—)	0 (—)	0 (—)	0 (—)	0 (—)
Apr–Jun	105 (11)	6 (11)	9 (20)	17 (40)	1 (3)	3 (18)
Jul–Sep	794 (82)	41 (75)	29 (64)	15 (35)	36 (95)	14 (82)
Oct–Dec	65 (7)	8 (15)	7 (16)	11 (26)	1 (3)	0 (—)
**Clinical syndrome**						
Nonneuroinvasive	338 (35)	7 (13)	20 (44)	4 (9)	0 (—)	2 (12)
Neuroinvasive	633 (65)	48 (87)	25 (56)	39 (91)	38 (100)	15 (88)
Encephalitis^†^	361 (57)	37 (77)	14 (56)	29 (74)	36 (95)	9 (60)
Meningitis^†^	215 (34)	10 (21)	4 (16)	5 (13)	2 (5)	4 (27)
AFP^†,§^	16 (3)	0 (—)	1 (4)	1 (3)	0 (—)	0 (—)
Unspecified^†^	41 (6)	1 (2)	6 (24)	4 (10)	0 (—)	2 (13)
**Outcome**						
Hospitalization	662 (68)	54 (98)	26 (58)	38 (88)	38 (100)	16 (94)
Death	60 (6)	0 (—)	2 (4)	9 (21)	19 (50)	0 (—)

**Abbreviation:** AFP = acute flaccid paralysis.

* Four unspecified California serogroup virus cases were also reported and are not shown.

^†^ Percentages of cases of encephalitis, meningitis, AFP, and unspecified neurologic presentations are percentages of neuroinvasive cases.

^§^ Among the 16 West Nile virus disease cases in persons with AFP, 10 (63%) also had encephalitis or meningitis.

Among the 633 WNV neuroinvasive disease cases, 361 (57%) were reported as encephalitis, 215 (34%) as meningitis, 16 (3%) as AFP, and 41 (6%) as unspecified neurologic signs or symptoms. A total of 584 (92%) patients with neuroinvasive disease were hospitalized, and 60 (10%) died. The median age of patients who died was 73 years (IQR = 67–82 years). The national incidence of neuroinvasive disease was 0.19 per 100,000 population (Table 2). The highest incidences occurred in Arizona (1.81 per 100,000), New Mexico (1.43), DC (1.28), and Nevada (1.10) (Figure). The largest numbers of neuroinvasive disease cases were reported from California (147), Arizona (132), Colorado (52), and Nevada (34), which together accounted for 58% of all neuroinvasive disease cases. The incidence of WNV neuroinvasive disease increased with age, from 0.01 per 100,000 in children aged <10 years to 0.55 in adults aged ≥70 years. Incidence was higher among males (0.24 per 100,000) than among females (0.14).

**TABLE 2:** Number and rate* of reported cases of arboviral neuroinvasive disease, by virus type, U.S. Census division, and state — United States, 2019

U.S. Census division/ State	Virus type, no. (rate*)					
	West Nile	La Crosse	Jamestown Canyon	Powassan	Eastern equine encephalitis	St. Louis encephalitis
**United States**	**633 (0.19)**	**48 (0.01)**	**25 (0.01)**	**39 (0.01)**	**38 (0.01)**	**15 (<0.01)**
**New England**	3 (0.02)	1 (0.01)	6 (0.04)	19 (0.13)	19 (0.13)	—^†^
Connecticut	1 (0.03)	—	—	5 (0.14)	4 (0.11)	—
Maine	—	—	—	2 (0.15)	—	—
Massachusetts	2 (0.03)	—	3 (0.04)	9 (0.13)	12 (0.17)	—
New Hampshire	—	—	3 (0.22)	2 (0.15)	—	—
Rhode Island	—	1 (0.09)	—	1 (0.09)	3 (0.28)	—
Vermont	—	—	—	—	—	—
**Middle Atlantic**	25 (0.06)	—	—	8 (0.02)	4 (0.01)	—
New Jersey	6 (0.07)	—	—	4 (0.05)	4 (0.05)	—
New York	14 (0.07)	—	—	4 (0.02)	—	—
Pennsylvania	5 (0.04)	—	—	—	—	—
**East North Central**	40 (0.09)	23 (0.05)	8 (0.02)	5 (0.01)	11 (0.02)	—
Illinois	22 (0.17)	—	1 (0.01)	—	—	—
Indiana	4 (0.06)	—	—	—	1 (0.01)	—
Michigan	11 (0.11)	1 (0.01)	1 (0.01)	—	10 (0.10)	—
Ohio	3 (0.03)	19 (0.16)	—	—	—	—
Wisconsin	—	3 (0.05)	6 (0.10)	5 (0.09)	—	—
**West North Central**	33 (0.15)	1 (<0.01)	11 (0.05)	7 (0.03)	—	—
Iowa	1 (0.03)	—	—	—	—	—
Kansas	7 (0.24)	—	—	—	—	—
Minnesota	2 (0.04)	1 (0.02)	11 (0.20)	6 (0.11)	—	—
Missouri	4 (0.07)	—	—	—	—	—
Nebraska	17 (0.88)	—	—	—	—	—
North Dakota	2 (0.26)	—	—	1 (0.13)	—	—
South Dakota	—	—	—	—	—	—
**South Atlantic**	33 (0.05)	10 (0.02)	—	—	2 (<0.01)	—
Delaware	—	—	—	—	—	—
District of Columbia	9 (1.28)	—	—	—	—	—
Florida	2 (0.01)	—	—	—	—	—
Georgia	9 (0.08)	1 (0.01)	—	—	1 (0.01)	—
Maryland	6 (0.10)	—	—	—	—	—
North Carolina	1 (0.01)	6 (0.06)	—	—	1 (0.01)	—
South Carolina	2 (0.04)	—	—	—	—	—
Virginia	4 (0.05)	—	—	—	—	—
West Virginia	—	3 (0.17)	—	—	—	—
**East South Central**	23 (0.12)	13 (0.07)	—	—	2 (0.01)	—
Alabama	4 (0.08)	—	—	—	1 (0.02)	—
Kentucky	4 (0.09)	1 (0.02)	—	—	—	—
Mississippi	12 (0.40)	—	—	—	—	—
Tennessee	3 (0.04)	12 (0.18)	—	—	1 (0.01)	—
**West South Central**	48 (0.12)	—	—	—	—	1 (<0.01)
Arkansas	7 (0.23)	—	—	—	—	—
Louisiana	11 (0.24)	—	—	—	—	—
Oklahoma	6 (0.15)	—	—	—	—	1 (0.03)
Texas	24 (0.08)	—	—	—	—	—
**Mountain**	272 (1.09)	—	—	—	—	8 (0.03)
Arizona	132 (1.81)	—	—	—	—	8 (0.11)
Colorado	52 (0.90)	—	—	—	—	—
Idaho	5 (0.28)	—	—	—	—	—
Montana	3 (0.28)	—	—	—	—	—
Nevada	34 (1.10)	—	—	—	—	—
New Mexico	30 (1.43)	—	—	—	—	—
Utah	14 (0.44)	—	—	—	—	—
Wyoming	2 (0.35)	—	—	—	—	—
**Pacific**	156 (0.29)	—	—	—	—	6 (0.01)
Alaska	—	—	—	—	—	—
California	147 (0.37)	—	—	—	—	6 (0.02)
Hawaii	—	—	—	—	—	—
Oregon	6 (0.14)	—	—	—	—	—
Washington	3 (0.04)	—	—	—	—	—

* Cases per 100,000 population, based on July 1, 2018, U.S. Census population estimates.

^†^ Dashes indicate no cases reported.

**FIGURE:**
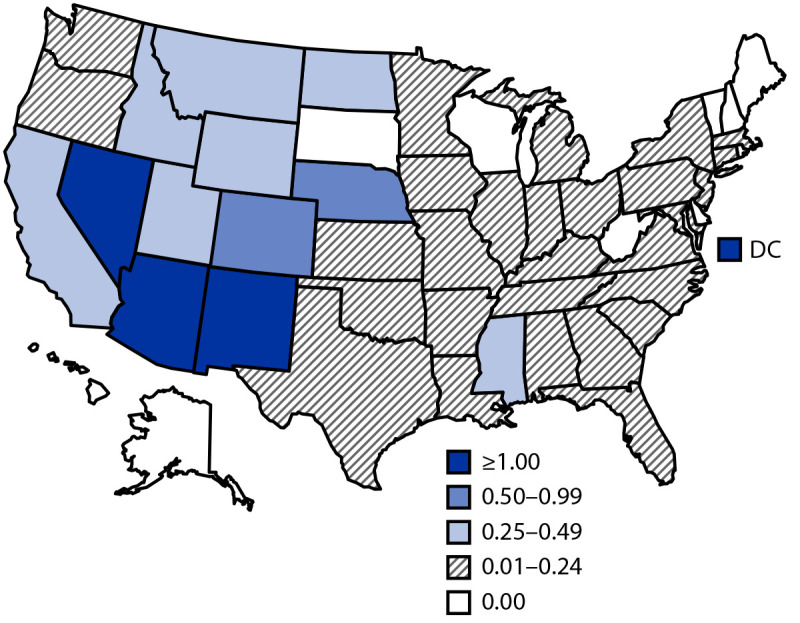
Incidence* of reported cases of West Nile virus neuroinvasive disease — United States,^†^ 2019 **Abbreviation:** DC = District of Columbia. * Cases per 100,000 population. ^†^ No cases were reported from Alaska or Hawaii.

Fifty-five La Crosse virus disease cases were reported from 10 states, with the highest number of cases reported from Ohio, Tennessee, and North Carolina (Table 2). The median patient age was 8 years (IQR = 5–12 years), and 51 (93%) were aged <18 years (Table 1). Thirty-three (60%) patients were male. Illness onset dates ranged from June to October, with 41 (75%) occurring during July–September. Forty-eight (87%) cases were neuroinvasive, and 54 (98%) patients were hospitalized; none died.

Forty-five Jamestown Canyon virus disease cases were reported from six states, with the highest number of cases reported from Minnesota and Wisconsin (Table 2). A disease case was reported for the first time from Illinois; however, the patient had traveled during the likely period of infection. The median patient age was 59 years (IQR = 31–70 years); 30 (67%) were male (Table 1). Illness onset ranged from April to November, with 29 (64%) cases occurring during July–September. Twenty-five (56%) cases were neuroinvasive, 26 (58%) patients were hospitalized, and two (4%) died, both aged 25–35 years and both with neuroinvasive disease.

Forty-three Powassan virus disease cases were reported from 10 states, with the highest number of cases reported from Massachusetts, Minnesota, and Wisconsin (Table 2). The median patient age was 64 years (IQR = 47–71 years); 31 (72%) were male (Table 1). Illness onset dates ranged from April to December, with 17 (40%) occurring during April–June. Thirty-nine (91%) cases were neuroinvasive. Thirty-eight (88%) patients were hospitalized. Nine (21%) patients died (all with neuroinvasive disease), eight (89%) of whom were aged >60 years.

Thirty-eight cases of eastern equine encephalitis virus disease were reported from 10 states. Twenty-two (58%) cases were reported from Massachusetts (12) and Michigan (10) (Table 2); cases were reported for the first time from Indiana and Tennessee. The median patient age was 64 years (IQR = 54–72 years); 27 (71%) were male. Illness onset dates ranged from June to November, with 36 (95%) occurring during July–September. All cases were neuroinvasive, and all patients were hospitalized. Nineteen (50%) patients died, all of whom were aged >50 years.

Seventeen cases of St. Louis encephalitis virus disease were reported from four states (Table 2). The median patient age was 65 years (IQR = 54–76 years); 12 (71%) were male (Table 1). Illness onset dates ranged from May to September, with 14 (82%) occurring during July–September. Fifteen (88%) cases were neuroinvasive; 16 (94%) patients were hospitalized, and none died.

## Discussion

As in previous years, WNV was the most common cause of domestic arboviral neuroinvasive disease in 2019. However, WNV neuroinvasive disease incidence (0.19 per 100,000) was 53% lower than the median annual incidence during 2009–2018 (0.40; range = 0.13–0.92) (*4*). The decrease in incidence was most notable in Midwestern and South Central states, particularly Texas, which reported 24 neuroinvasive disease cases, 78% lower than its annual median of 111 (range = 20–844) during 2009–2018 (*4*). Despite overall low WNV disease incidence, multiple states reported more cases than their annual median during 2009–2018, mostly in the Mountain region (*4*).

La Crosse virus continued to be the most common cause of neuroinvasive arboviral disease in children (*5*). Jamestown Canyon virus disease incidence has increased over time, with a median of 45 (range = 41–75) cases reported annually during 2017–2019 compared with 11 (range = 0–22) during 2010–2016 (*6*). More cases of Powassan virus disease were reported for 2019 than any previous year, with 43 cases compared with the previous high of 34 cases in 2017 and a median of 15 cases annually during 2010–2018 (*7*). More cases of eastern equine encephalitis virus disease were reported for 2019 (38) than for any previous year; the previous high of 21 cases was reported in 2005, and a median of seven cases was reported each year during 2010–2018 (*8*). Eastern equine encephalitis virus remained the deadliest arbovirus disease, with one half of patients dying. For viruses with higher than average case numbers in 2019, whether the increase reflects an actual increase in disease incidence or increased awareness, surveillance, and testing is unknown.

Although the reported number of cases varies, arboviruses cause substantial morbidity in the United States each year. Cases occur sporadically, with epidemiology varying by virus and geography. Weather, zoonotic host, vector abundance, and human behavior all influence when and where arboviral outbreaks occur, making it difficult to predict locations and timing of cases and underscoring the importance of surveillance in identifying outbreaks and informing prevention efforts.

The findings in this report are subject to at least two limitations. First, arboviral diseases are likely underrecognized and underreported to ArboNET. This is especially true for nonneuroinvasive disease; previous studies estimated that 30 to 70 nonneuroinvasive cases occur for every neuroinvasive WNV disease case reported (*9*). Based on the 633 neuroinvasive WNV disease cases reported for 2019, 18,990 to 44,310 nonneuroinvasive WNV disease cases could have occurred; however, only 338 (1%–2%) were reported. Second, because ArboNET does not require information about clinical signs, symptoms, or laboratory findings, cases might be misclassified.

Health care providers should consider arboviral infections in cases of aseptic meningitis and encephalitis, obtain necessary specimens for laboratory testing, and promptly report cases to public health authorities (*2*,*3*). Understanding the epidemiology, seasonality, and geographic distribution of these arboviruses is important for clinical recognition and differentiation from other neurologic infections. Because human vaccines against domestic arboviruses are not available, prevention depends on community and household efforts to reduce vector populations (e.g., applying insecticides and reducing breeding sites), use of personal protective measures to decrease mosquito and tick exposures (e.g., repellents and protective clothing), and blood donation screening to minimize alternative routes of transmission.

**Summary**

**What is already known about this topic?**

West Nile virus (WNV) is consistently the leading cause of domestically acquired arboviral disease, but other arboviruses cause sporadic cases and outbreaks of neuroinvasive disease, resulting in substantial morbidity and mortality.

**What is added by this report?**

In 2019, WNV neuroinvasive disease incidence was 53% lower than the median annual incidence during 2009–2018. More Powassan and eastern equine encephalitis virus disease cases were reported than in any previous year.

**What are the implications for public health practice?**

Health care providers should consider arboviral infections in patients with aseptic meningitis or encephalitis, perform recommended diagnostic testing, and promptly report cases to public health authorities. Surveillance is important to identify outbreaks and guide prevention strategies, which include wearing insect repellent, long pants, and long-sleeved shirts when outdoors.

## Footnotes

*
https://www.cdc.gov/ncezid/dvbd/media/stopmosquitoes.html

†
https://www.cdc.gov/ticks/avoid/index.html
