# *Erratum:* Vol. 70, No. 32

**DOI:** 10.15585/mmwr.mm7034a6

**Published:** 2021-08-27

In the report “West Nile Virus and Other Domestic Nationally Notifiable Arboviral Diseases — United States, 2019,” on pages 1072 and 1073, in Table 2, rates should have been **<0.01** for the following viruses and U.S. Census divisions: La Crosse virus for the West North Central Division; Eastern equine encephalitis for the South Atlantic division; and St. Louis encephalitis for the United States (total) and the West South Central division.

